# Assessment of physician preparedness for implementation of pathology-supported genetic testing: solution-driven post-COVID-19 survey

**DOI:** 10.3389/fgene.2025.1543056

**Published:** 2025-03-21

**Authors:** Elouise E. Kroon, Yolandi Swart, Chantelle J. Scott, Denise Scholtz, Daniel W. Olivier, Kelebogile E. Moremi, Chantelle Venter, Maxine Waters, Sunday O. Oladejo, Craig J. Kinnear, Etheresia Pretorius, Kanshukan Rajaratnam, Desiree C. Petersen, Marlo Möller, Maritha J. Kotze

**Affiliations:** ^1^ South African Medical Research Council Centre for Tuberculosis Research, Division of Molecular Biology and Human Genetics, Faculty of Medicine and Health Sciences, Stellenbosch University, Cape Town, South Africa; ^2^ Department of Pathology, Division of Chemical Pathology, Faculty of Medicine and Health Sciences, Stellenbosch University, Cape Town, South Africa; ^3^ Division of Chemical Pathology, Department of Pathology, National Health Laboratory Service, Tygerberg Hospital, Cape Town, South Africa; ^4^ Department of Physiological Sciences, Faculty of Science, Stellenbosch University, Stellenbosch, South Africa; ^5^ School for Data Science and Computational Thinking, Stellenbosch University, Cape Town, South Africa; ^6^ South African Medical Research Council Genomics Platform, Cape Town, South Africa; ^7^ Department of Biochemistry, Cell and Systems Biology, Institute of Systems, Molecular and Integrative Biology, Faculty of Health and Life Sciences, University of Liverpool, Liverpool, United Kingdom; ^8^ National Institute for Theoretical and Computational Sciences (NITheCS), Stellenbosch, South Africa; ^9^ Centre for Bioinformatics and Computational Biology, Stellenbosch University, Stellenbosch, South Africa

**Keywords:** COVID-19, education, genetic counseling, genomics, non-communicable disease (NCD), pathology-supported genetic testing (PSGT), survey, wellness

## Abstract

**Introduction:**

Rapid advances in personalized medicine and direct-to-consumer genomic applications could increase the risk that physicians will apply genomic results inappropriately. To address a persistent lack of understanding of genomics, we implemented a pathology-supported genetic testing (PSGT) approach, guided by insights from a clinician needs assessment conducted in 2010.

**Methods:**

Findings from the previous clinician survey were used to develop a new patient screening tool that integrates non-communicable disease (NCD) and post-COVID-19 care pathways. In parallel to the application of this solution for stratification of patients in different treatment groups, an updated version of the original survey questionnaire was used to reassess the knowledge and willingness of healthcare professionals to apply PSGT.

**Results:**

Thirty-six respondents completed the revised needs assessment survey in October 2022, while attending a genomics session at the Annual General Practitioner Congress, Stellenbosch University, South Africa. Nearly 89% of the respondents reported having insufficient knowledge to offer genetic testing; 80% were supportive of using PSGT to differentiate inherited from lifestyle- or therapy-associated NCDs and 83.3% supported integrating wellness screening with genetic testing to identify high-risk individuals.

**Discussion:**

It appears that while clinicians are interested in learning about genomics, they continue to report significant knowledge deficits in this area, highlighting the need for targeted clinician training and tools like multidisciplinary NCD-COVID pathway analysis to improve clinical decision-making. The co-development of a genomic counseling report for ongoing studies, guided the selection of Long COVID patients for whole-genome sequencing across the illness and wellness domains.

## 1 Introduction

The rapid development of molecular genetic technologies and targeted therapies necessitates increased knowledge and awareness of these modalities among healthcare practitioners, serving as intermediaries between the public and genetic testing laboratories (Kotze et al., 2015). However, most primary healthcare practitioners are inexperienced and lack confidence in requesting genetic tests for their patients (Harding et al., 2019). This knowledge gap hinders patient referral, despite calls for improved genetic literacy since the nineties (Hunter et al., 1998). Continued education and development of decision support systems are therefore crucial to enable personalised patient care (Wonkam et al., 2006; de Abrew et al., 2014).

Questionnaire-based surveys are an ideal way to gauge practitioner knowledge and to identify disagreement gaps regarding available genetic/genomic tests that need to be addressed (Carroll et al., 2019; Crellin et al., 2019). In a 2015 survey distributed to 150 general practitioners attending a family practitioners’ conference in South Africa, more than half of the 51 respondents rated their knowledge of genetics as insufficient (Düsterwald, 2015). Down syndrome, followed by cystic fibrosis and breast cancer, were reported to be the most frequent genetic disorders encountered in primary care.

Precision oncology paved the way in bridging the gap between personalised medicine and generalized primary care, as demonstrated for *BRCA1/2* genetic testing performed in parallel with *CYP2D6*-pharmacogenomics and transcriptional gene profiling (MammaPrint) (Baatjes et al., 2017; Mampunye et al., 2021). In this context, Kotze et al. (2024) demonstrated the value of pathology-supported genetic testing (PSGT) to navigate ethical challenges anticipated with the implementation of genomic medicine in South Africa. Breast cancer genomics illustrated that what seems to be a single disease comprises at least four major tumor subtypes, each requiring different treatment strategies. A multi-omics approach is also advocated by Wang et al. (2023), to fulfill the promise of precision medicine defined as giving the right treatment at the right time to the right patient (Caudle et al., 2014; Bycroft et al., 2018; Jorgensen et al., 2019).

The present study was adapted from and builds on the results of a physician needs assessment survey, which demonstrated significant underutilization of genetic services in South Africa as well as support for PSGT (Vogel et al., 2011). Given the vast changes and increased public requests for genetic testing and services, we wanted to understand if medical practitioners felt more equipped and open to genetic testing than in the past. Rather than focusing on a comparison, we wanted to gain a snapshot into medical practitioners’ present mindset and thoughts around genetic testing, specifically when requested within the context of non-communicable diseases (NCDs). The interpretation of genetic variants within the context of NCD risk profiles can be tricky to navigate, especially within the framework of low penetrance variants. The intended meaning and clinical relevance of laboratory results reported outside the context of an individual’s unique clinical risk profile may be lost in translation. PSGT was developed to cross this bridge. Genetic and environmental risk factors as well as familial data are drawn into the clinical realm and correlated with relevant biochemical parameters to translate population risk into personal utility. While public health genomics aims to meet the increasing demands of serving the greater good vs. the risk-benefit gain of a personalized medicine approach (Zimmern and Khoury, 2012; Moyo et al., 2023), PSGT combines both concepts to provide a comprehensive understanding of health and disease (Kotze et al., 2013).

Jongeneel et al. (2022) published a checklist with key requirements for assessing the readiness for implementation of personalized genomic medicine programs in Africa. This includes upskilling of healthcare workers to produce an interdisciplinary workforce for research translation enabled by the availability of online platforms for practicing implementation ideas such as PSGT. With emergence of the coronavirus in 2019 (COVID-19), significant investments in infrastructure, sequencing technologies and training were applied effectively to catapult developing continents, such as Africa, to the frontline as world leaders in genomic surveillance (Tegally et al., 2022). A shift from paper-based to electronic informed consent for PSGT created the opportunity to bring well-established human genetics principles into COVID-19 risk management (Daramola et al., 2021). Towards this end, commonalities identified among patients with NCDs also implicated in COVID-19 severity (Pretorius et al., 2022; Dissanayake, 2023), could be incorporated into a wellness screening tool for assessment of genomic testing eligibility. During this period a mobile data capturing app was developed, with the aim of connecting patients to the appropriate medical providers, including genetic counsellors and medical practitioners. Individualized NCD-COVID pathway reports integrating the effect of environmental risk factors on expression of genetic variants selected for testing, are correlated with blood biochemistry results to identify individuals who may benefit from comprehensive whole genome sequencing (WGS) and genetic counselling across the illness and wellness domains. We provide a case example of the successful implementation of this concept in a patient.

The current survey questionnaire underscores the present need for a clinician education program that provides hands-on PSGT training to better equip medical practitioners to implement genetic testing within the scope of their practice, with tools such as mobile data capturing apps to streamline the process.

## 2 Materials and methods

### 2.1 Ethics approval

Ethics approval for the survey was obtained from the Health Research Ethics Committee (HREC), Stellenbosch University (N22/07/082). We expanded a questionnaire that forms part of a translational research project, focused on the development of personalized medicine solutions and return of research results to patients using an integrated service and research approach (N22/11/133).

### 2.2 Study design for data collection

The study followed a cross-sectional design using a mixed-methods model. Registered medical practitioners attending the annual general practitioner conference hosted by Stellenbosch University in October 2022 were invited to complete the online survey. The questionnaire consisted of 5 sections (A-E), which introduced the PSGT concept (A,B) towards a readiness assessment (C,D) based on the uptake of genetic counselling and testing services (E) by general practitioners in South Africa.

Table 1 summarizes the survey objectives to determine the clinical usefulness of a wellness screening tool developed in response to the COVID-19 pandemic.

**TABLE 1:** Study objectives for a solution-driven post COVID-19-survey.

Survey themes reported on in 2011	Modified survey focused on in 2022
A: 6 questions Perception of personalized medicine and availability of resources to ensure continuous clinician education in genomic applications	Our objective 1 was to use section A to conduct an overall review of the current clinician views and perceptions of personalized medicine (Table 3)
B: 9 questions Benefit to patients using genetic testing either as a disease prevention or treatment modality	For objective 2 we wanted to understand if clinicians practicing in the COVID-19 era felt that genetic testing may be useful to identify individuals with comorbidities at higher risk for infectious diseases. (Supplementary Table S1)
C: 10 questions Clinical usefulness of selected genetics tests in GP practice including the final assessment of the “*pathology supported genetic test* (PSGT) including components of specified assays forming part of a wellness program that may be offered by medical schemes”.	Objective 3 was to determine if the clinicians deemed there was value in a PSGT approach incorporating different tests for non-communicable diseases such as breast cancer, cardiovascular and depression into an adaptable patient report (Supplementary Table S2)
D: 10 questions Usefulness of online tools for test requests and motivation for medical aid reimbursement based on clinical characteristics disclosed for medical aid pre-authorization	Not assessed, as proof of concept was already demonstrated for the 70-gene MammaPrint tumour genomics assay reimbursed as part of oncology benefits by many medical schemes in SA, following a health technology assessment of PSGT in the context of breast cancer (Stage I and II) recurrence risk (Grant et al., 2013)
E: 10 questions Need for future workshops as well as the future intended use of genetic services, including testing and counselling modalities	Our overall objective was to assess the need for further education and perceived value of genetic counselling with an illustrative example, which can be incorporated as a training tool in clinical practice

### 2.3 Procedures

Participants in the study were asked to rate their opinion pertaining to the interdisciplinary PSGT methodology incorporating components of individual genetic assays that may be included as part of a wellness program offered by medical schemes. Previous use of genetic services prior to completion of the questionnaire, or attendance of the workshop in a subset of the respondents, was compared with future willingness to incorporate genetic services into clinical practice. All statements relating to genetic testing were evaluated according to a 5-point Likert scale, i.e. (1 = strongly agree; 2 = agree; 3 = neutral; 4 = disagree to 5 = strongly disagree).

The questionnaire was uploaded and linked to a secure database, RedCap (Harris et al., 2009; Harris et al., 2019). Participants provided online informed consent to participate. The entire survey was anonymous. Participants could complete the questionnaire during and after the GP conference.

### 2.4 Data analysis

Data was analyzed in RStudio (RStudio Team, 2020). Responses for “strongly agree” and “agree,” as well as “disagree” and “strongly disagree” were combined to either “agree” or “disagree.” The frequency of responses for each category “agree,” neutral or “disagree” were calculated for each question. Thereafter, the frequency was expressed as a percentage of the total number of participants n = 36.

### 2.5 Development of post-COVID clinician education tools

Based on the clinician survey results, we propose developing educational tools centered on case presentations in genomic counseling reports. Practically, these tools will incorporate new insights gained during the COVID-19 pandemic, following the removal of cost barriers for medical scheme beneficiaries in the pilot genomics access program described by de Klerk et al. (De Klerk et al., 2024). The same training platform has been contextualized for managing patients with prolonged symptoms after the diagnosis of COVID-19 that requires a broader training strategy than cascade testing traditionally used to assess familial risk (Pretorius et al., 2022). The practical clinical implications of this new knowledge will require the stratification of patients into different treatment groups integrating genetic data, microclot pathology findings, and advanced data science methods to enable pattern recognition through both illness and recovery phases. This concept is illustrated in the discussion section for implementation in accordance with the roadmap for Long COVID data sharing (Oladejo et al., 2023).

## 3 Results

A total of 36 healthcare practitioners completed the survey. Thirty-two were general practitioners (GPs) and four persons indicated their profession as ‘other’ without any further specification (Table 2). By indicating their profession as ‘other’ it may include any medical healthcare practitioner other than GPs, cardiologists, dieticians, gynecologists, oncologists and surgeons. Of the participants 67% (24/36) were female and 33% (12/36) male (Table 2). Most participants (42%, 15/36) were aged 35–44 years (Table 2).

**TABLE 2:** Survey respondent characteristics.

Gender	
Female	24/36 (67%)
Male	12/36 (33%)

Age	
25–34	5/36 (14%)
35–44	15/36 (42%)
45–54	10/36 (28%)
55–64	5/36 (14%)
65 and older	1/36 (2.8%)

Profession	
GP	32/36 (89%)
Other	4/36 (11%)

For objective 1, participants viewed personalized medicine as the ability to identify subgroups of patients that differ in their genetic susceptibility to a particular disease and/or their response to a specific intervention (75%, 27/36). Sixty-nine percent (25/36) of participants agreed that genetic alterations are permanent and that their expression can sometimes be modified by environmental factors (e.g., diet, medication) to increase or decrease risk. Sixty-six percent (24/36) also agreed that integration of health services would improve the current model of molecular genetics as a stand-alone laboratory service. In addition, sixty-four percent (23/36) recognized the need for the appropriate clinical indications and individualized medical supervision to avoid compromising patient health when offering genetic tests providing health-related information (Table 3).

**TABLE 3:** Section A results.

SECTION A			
1 = agree; 2 = neutral; 3 = disagree	1	2	3
There is a shortage of genetic counsellors and clinicians trained in genetics	30/36 (83%)	4/36 (11%)	2/36 (6%)
The increase in the number and complexity of genetic tests necessitates regular educational updates for clinicians	29/30 (80%)	5/36 (14%)	2/36 (6%)
Personalized medicine refers to the ability to identify subgroups of patients that differ in their genetic susceptibility to a particular disease and/or their response to a specific intervention	27/36 (75%)	7/36 (19%)	2/36 (6%)
Genetic alterations are permanent, but their expression can sometimes be modified by environmental factors (e.g., diet, medication) to increase or decrease risk	25/36 (69%)	6/36 (17%)	5/36 (14%)
The current model of molecular genetics as a stand-alone laboratory service can be improved by integration with other health disciplines	24/36 (67%)	9/36 (25%)	3/36 (8%)
The offer of genetic tests providing health-related information in the absence of clinical indications and individualized medical supervision may compromise patient health	23/36 (64%)	6/36 (17%)	7/36 (19%)

In section B, objective 2, sixty-four percent (23/36) indicated that genetic testing could be useful to identify individuals with comorbidities at higher risk for infectious diseases, with around seventeen percent (6/36) of responders showing no interest in such a genetic risk stratifying approach (Supplementary Table S1). Similar to the previous survey, most participants (81%, 29/36) agreed that a genetic test is most meaningful when it can be used in a clinical context to simultaneously 1) diagnose treatable disease subtypes, 2) prevent cumulative risk, and 3) formulate an individualized treatment plan (Supplementary Table S3, objective 3). This relates to sixty-one percent of participants who agreed that a PSGT approach including components of the individual tests questioned in section C (Supplementary Table S2) could be combined into a wellness program offered by medical schemes, possibly extended to incorporate host-genetics into NCD-COVID pathway reports.

Most participants (83%, 30/36) indicated that there is currently a shortage of genetic counsellors and clinicians trained in genetics, with the increase in the number and complexity of genetic testing necessitating the need for regular educational updates for clinicians (81%, 29/36) (Table 3). Despite most participants recognizing the need for genetic counsellors and further training (86%, 31/36 were interested in future workshops to improve genetic knowledge), only 41.7% (15/36) of participants requested genetic tests for their patients in the past while only 11.1% (4/36) felt that they had sufficient knowledge to request genetic tests (Table 4). Only 33% (12/36) have previously referred patients for genetic counselling to enable them to make their own free decisions about genetic testing, however most participants (94%, 34/36) intended to refer patients for genetic counselling by a registered genetic counsellor in the future. The 5/36 participants who were not interested in future genetic education opportunities, did not feel that they had sufficient genetic knowledge (100%, 5/5), have never requested previous genetic testing or referred a patient to a genetic counsellor (100%, 5/5), but were all willing to refer in the future (100%, 5/5) (Table 4).

**TABLE 4:** Section E results.

SECTION E		
The following questions will assess the use of genetic testing and counselling	YES	NO
Have you requested genetic testing for your patients in the past?	21/36 (58%)	15/36 (42%)
Have your patients benefited (e.g., early diagnosis) from genetic testing in the past?	18/36 (50%)	18/36 (50%)
Will you request genetic testing performed at an accredited laboratory for your patients in future?	30/36 (83%)	6/36 (17%)
Have you referred patients for genetic counselling in the past to enable them to make their own free decisions about genetic testing?	12/36 (33%)	24/36 (67%)
Will you refer patients for counselling by a registered genetic counsellor in future?	34/36 (94%)	2/36 (6%)
Will you prefer to use a secure online computer program that provides all the relevant information to request genetic testing and/or counselling for your patients?	31/36 (86%)	5/36 (14%)
Do you feel like you have sufficient genetic knowledge to request genetic tests for your patients?	4/36 (11%)	32/36 (89%)
Would you be interested in workshops aimed at improving your genetic knowledge?	31/36 (86%)	5/36 (14%)
Would you be interested in following social media platforms with regards to the latest genetic developments?	25/36 (69%)	11/36 (31%)

Eighty-three percent (30/36) of the participants were willing to request future genetic tests from an accredited laboratory, with the greater majority (86%, 31/36) recognizing the value of technology and preferring to use an online secure computer program providing relevant information to request genetic testing/genetic counselling for their patients (Table 3).

## 4 Discussion

Ten years after the original survey, the current results obtained did not demonstrate any improvement in the uptake of well-established germline DNA tests for monogenic diseases and genetic counselling in GP practice. In the previous survey, 76.9% (140/182) of healthcare professionals were willing to refer their patients to genetic counsellors. However, this did not happen to the extent indicated, as reflected in our follow-up survey, with only 33% (12/36) of healthcare practitioners having used the service of a genetics counsellor. Clinicians who were not interested in further training, likely realized their limited capacity in busy healthcare practices and expressed an interest to refer their patients in future to genetic counsellors.

Support for the PSGT approach remained high in the current survey and justified the development of three novel technologies over the last decade, specifically designed to bridge the clinical implementation gaps identified in the first clinician needs assessment survey. These include an online application enabling a wellness survey to capture family history, lifestyle factors and medication usage; point-of-care genetic testing to increase access to genomic medicine; and a semi-automated reporting system for integration of these diverse data sets with pathology test results to facilitate clinical interpretation. Utilisation of these technologies as educational tools in the COVID-19 era, was preceded by proof of principle studies that demonstrated the clinical utility of PSGT to prevent misdirection and fragmentation of genetic services in breast cancer risk management (Grant et al., 2013; Mampunye et al., 2021; Okunola et al., 2023).

Given the general lack of genomic knowledge, survey respondents agreed that incorporating different assays for NCDs such as breast cancer, cardiovascular and depression into the PSGT platform, may be advantageous. This has now become a reality with advances in next-generation sequencing using whole-exome and whole-genome sequencing (WES/WGS) (Kroon et al., 2025). Given the fact that 6% of medical practitioners disagreed that *BRCA1/2* gene screening is useful, even though it is a well-established clinical approach to breast cancer, it is encouraging that only 14% disagree with a PSGT approach (see responses in Supplementary Table S2). This indicates that we must acknowledge the reality that not everyone is aware of the potential benefits to patients, irrespective of utilising stand-alone genetic tests or an integrated genomics approach.

Direct to consumer testing (DTC) has seen increasing clientele seeking to understand and guide their healthcare decisions (Dandara et al., 2013; Horton et al., 2019; Martins et al., 2022). Lack of inclusion of ancestry-specific variants in gene panels used outside of the contextualised content inherent to PSGT, has led to increased questioning of the value of DTC testing, especially in persons with mixed genetic ancestry. Globalization has contributed to more genetically diverse populations worldwide, with traditional social population group descriptors such as “race” becoming increasingly clinically redundant (Brett and Goodman, 2021; Kaufman et al., 2021). The stepwise PSGT approach may be an effective solution to identify possible causal variants, irrespective of self-identified race/ethnicity. This may be achieved by utilizing available point-of-care assays, incorporating common gene variants detected at an increased frequency in certain geographical areas due to pleiotropic or founder effects, before embarking on more expensive whole exome/genome sequencing in unresolved cases (De Klerk et al., 2024). The correct interpretation and the appropriate application of tests offered through DTC testing requires assessment of the clinical relevance of individual gene variants as enabled by the PSGT approach.

An integral aspect of patient feedback with genetic testing is the need for comprehensive counselling. This can be provided by a healthcare practitioner trained in genetics, but also by genetic counsellors (Biesecker, 2020), often viewed as the gatekeepers of genomic medicine. The integral value of genetic counsellors lies with their comprehensive patient approach and added value of longer consultation times in comparison to clinicians. Within a professional and ethical value framework, genetic counsellors incorporate a unique merging of skills, including written and verbal communication; critical thinking; interpersonal, counselling, and psychosocial assessment skills (Uhlmann et al., 2009). This framework is vital to provide emotional support to patients as it assists in a clearer understanding of medical conditions with the benefit of more informed choices, and ultimately, facilitate trust in the medical fraternity (Abacan et al., 2019). Within a South African context, the roles of genetic counsellors may even be extended to administration, public engagement, marketing, research, and teaching (Kromberg and Krause, 2013). Assisting genetic counsellors with this role includes the use of an integrated PSGT report to direct insightful feedback, counselling, and advice for clients.

Rapid technological developments over the last decade have increased practitioners’ access to genetic services. Challenges associated with the emergence of COVID-19 in 2019, accompanied by prolonged community distancing, enhanced the interpretative commenting skills through online patient data processing from sample collection to test result (Daramola et al., 2021). In this context, PSGT of NCD metabolic pathways provides a cost-saving approach prior to embarking on comprehensive next-generation sequencing technologies in uninformative cases. To this end, the PSGT data-driven platform provides a service to medical scientists collaborating closely with genetic counsellors as part of the research team to translate genomic research into clinical practice. A new mobile phone app was launched from the platform (Kotze et al., 2023), and first introduced at the annual general practitioner conference hosted by Stellenbosch University in October 2022 when GPs were invited to complete the current survey. A suggested new addition for the app is to provide clinicians trained in genetics the option to complete any pre- or post-test counselling themselves or to prompt referral to genetic counsellors if they are not comfortable to conduct genetic counselling. The role of the genetic counsellor in these instances would be to assess the urgency and appropriateness of PSGT within the clinical management context of each online referral. Only once post-test genetic/genomic counselling is complete, can the report be released to the patient. Using this integrated approach during student training in research translation programs involving a multi-disciplinary team, will enhance the implementation of personalized healthcare.

### 4.1 Practice implications

The above-mentioned activities were performed in accordance with the requirements of the Protection of Personal Information Act (POPIA) in South Africa. Key to such a platform is the integration of comprehensive personal patient information (including lifestyle and personal and family medical history) with clinical pathology and genetic test results, for separate sections in the feedback report directed to the healthcare practitioner and patient. Within this framework genetic counsellors facilitate a key role in assisting medical practitioners in ensuring that patients understand the meaning and implications of the genetic tests and results. In turn, the semi-automated reports assist genetic counsellors, other healthcare practitioners and patients to implement personalized healthcare plans based on the integrated results.

**FIGURE 1:**
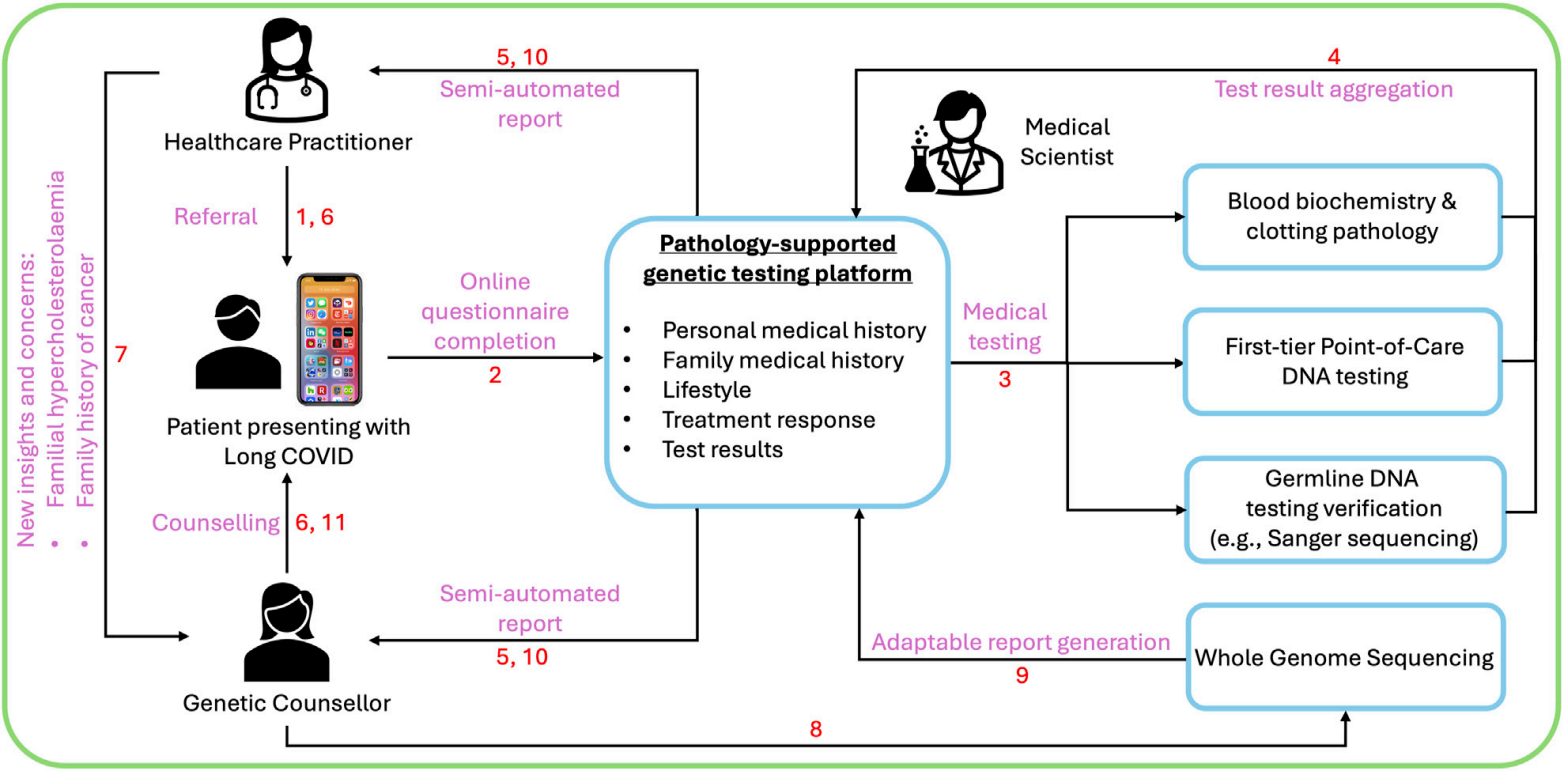
Novel genetic counseling concept developed in response to the COVID-19 pandemic using a pathology-supported genetic testing platform. This research translation and educational process is initiated by patient referral and/or participation in a wellness survey (1), facilitated by online informed consent for a questionnaire-based family history and health assessment and data sharing (2). Hereafter, the relevant medical and genetic testing is completed (3) and aggregated into a semi-automated NCD-COVID pathways report (4). This adaptable genomic counseling report is reviewed/edited by the multi-disciplinary healthcare team for discussion with the patient (5, 6). In cases where additional extended genetic testing is clinically indicated, the patient is referred for genetic counselling based on new diagnostic and pharmacogenomic insights unique to each patient (7), to discuss the option of extended genetic testing using advanced technologies such as whole genome sequencing (8). After completion of this comprehensive analysis the adaptable patient report is elevated to include the additional genetic data (9), which is reinterpreted in the context of the updated and previously collected clinical and first-tier genetic information (10). The results are discussed with the patient and the referring healthcare practitioner by the genetic counsellor (11).

Figure 1 showcases an example of genetic counselling offered to a patient with Long COVID, in line with the suggestion to use a single platform for information processing that extends from data collection to report generation (Oladejo et al., 2023). The patient opted for a wellness screen adopted for Long COVID based on the outcome of this study, given the cumulative risk imposed by metabolic syndrome features known to increase the risk for adverse COVID-19 outcomes (Pretorius et al., 2022; Dissanayake, 2023). A semi-automated report was generated for combined research and service delivery using the PSGT approach after the patient’s full personal, medical, family, diet and lifestyle history was captured. The patient did not have direct access to the data collection app to request genetic testing or the NCD-COVID pathway report resulting from the research, without referral from a healthcare practitioner. A multidisciplinary team structured the semi-automated report into recommendations for lifestyle adjustments, change of treatment and possible extended genetic testing using WGS (Figure 1). Genetic counselling was performed to determine eligibility for WGS, comparable to the rationale for first-tier genotyping of the same pleiotropic gene variants extended to WES, in a patient diagnosed with pulmonary embolism and warfarin resistance (Kroon et al., 2025).

While referred for PSGT of six NCD pathways as previously described by De Klerk et al. (2024), this approach identified the need for extended genetic testing for familial hypercholesterolemia, based on correlation with biochemistry results and consideration of the family history, which was reported in the online app-generated patient report. In addition, a strong family history of cancer provided an opportunity for further counselling due to the need for comprehensive cancer genetic testing to rule out inherited cancer syndrome. Beyond the recommended lifestyle and dietary interventions, this patient was alerted to further tests and counselling, which may benefit not only her, but also her children and their future offspring.

### 4.2 Study limitations

Due to a small sample size, statistical inference is limited. For this reason, we opted for a qualitative approach. Further genetic training for healthcare practitioners should be tailored to address the intricate relationship between genotype, phenotype, and environmental factors, which are essential for assessing risks from a broader population to an individual level. The underutilization of pathology in linking genes to diseases was addressed by PSGT recognized in 2020 by the International Consortium of Personalised Medicine as an example of best practice in cancer genomics. Accurate phenotyping is crucial not only in single gene disorders but also for complex NCDs (Kroon et al., 2025). Although the use of an unvalidated survey is a limitation, new insights gained from this study exemplified by the case example, opened new avenues for research and training beyond the limited objectives defined for this study. This includes the development of innovative technologies in parallel to standard methodologies.

## 5 Conclusion

The need for improved regulation and the support of an integrated multidisciplinary team for the request and feedback of genetic testing is becoming increasingly evident. To counteract the increasing demand for DTC testing despite harm caused by misleading information due to oversimplification of genomics as a complex science, the establishment of credible platforms is crucial to provide legitimate patient feedback and counselling services.

Despite educational workshops and free online availability of genetics resources, most medical practitioners still feel that they lack sufficient knowledge regarding genetics. Postgraduate training opportunities for clinicians are limited to those who opt to specialize in Clinical Genetics and exposure to human genetics for undergraduate medical students remains introductory. Undergraduate genetic teaching and development of access tools tailored to medical practitioners’ needs will increase the understanding and appropriate use of genomics in clinical practice. In addition, increased training and job opportunities need to be made available for genetic counsellors who play a critical role in the feedback, accurate interpretation and understanding of genetic results. Tools such as the semi-automated NCD-COVID pathway reports may assist counsellors to provide integrated feedback to patients, while guiding healthcare practitioners to apply the results to patient treatment plans.

We therefore propose the creation of educational tools focused on case presentations in genetic counseling reports. These tools will integrate family pedigrees with new knowledge for stratifying patients into different treatment groups. Ultimately, a combination of genetic and pathology data analyzed by advanced data science techniques will facilitate the extraction of meaningful information to improve risk management of both NCDs and communicable diseases.

## Data Availability

The original contributions presented in the study are included in the article/Supplementary Material, further inquiries can be directed to the corresponding author.
