# Supplementary material for: Assessment of physician preparedness for implementation of pathology-supported genetic testing: solution-driven post-COVID-19 survey
Source: Front Genet. 2025 Mar 21;16:1543056. doi: 10.3389/fgene.2025.1543056 (PMC11970434; doi:10.3389/fgene.2025.1543056)
Supplement: Supplementary file 1 [file DataSheet1.pdf]

Supplementary tables and figures:

Supplementary table 1: Section B results

SECTION B:			
**1=agree; 2=neutral; 3=disagree**	**1**	**2**	**3**
To diagnose a genetic disorder.	32/36 (89%)	1/36 (3%)	3/36 (8%)
To assess disease prognosis and recurrence risk.	27/36 (75%)	5/36 (14%)	4/36 (11%)
To identify genetic risk factors contributing to disease development.	27/36 (75%)	5/36 (14%)	4/36 (11%)
To prevent disease development in healthy individuals based on the family history.	24/36 (67%)	8/36 (22%)	4/36 (11%)
To identify subtypes of complex diseases to select the best treatment option.	30/36 (83%)	4/36 (11%)	2/36 (6%)
To identify high risk individuals who require aggressive treatment.	32/36 (89%)	1/36 (3%)	3/36 (8%)
**To identify individuals with comorbidities at higher risk for infectious diseases**	**23/36 (64%)**	**7/36 (19%)**	**6/36 (17%)**
To identify individuals most likely to benefit from diet and lifestyle changes only.	25/36 (70%)	7/36 (19%)	4/36 (11%)
To obtain information relating to more than one of the above in a single test.	23/36 (64)	7/36 (19%)	6/36 (17%)

Supplementary table 2: Section C results

**SECTION C:**				
**1=agree; 2=neutral; 3=disagree; 4=do not know this test**	**1**	**2**	**3**	**4**
BRCA1/2 gene screening to identify a high risk for familial breast cancer.	31/36 (86%)	3/36 (8%)	2/36 (6%)	NA
Identification of a *BRCA1/2* mutation to motivate the need for a (prophylactic) bilateral mastectomy in women who developed breast cancer at a young age.	30/36 (84%)	3/36 (8%)	3/36 (8%)	NA
The MammaPrint test to reduce the use of chemotherapy in patients with early-stage, estrogen receptor-positive breast cancer.	26/36 (72%)	6/36 (17%)	2/36 (6%)	2/36 (6%)
CYP2D6 genotyping to identify Tamoxifen resistance in breast cancer patients.	23/36 (64%)	10/36 (27%)	1/36 (3%)	2/36 (6%)
CYP2D6 genotyping to prevent side effects in 10-20% of individuals using anti-depressants and other commonly prescribed medications.	19/36 (53%)	12/36 (33%)	3/36 (8%)	2/36 (6%)
Mutation analysis of the *LDL* receptor and/or *ApoE* genes in patients with high cholesterol to choose the best treatment strategy.	22/36 (61%)	14/36 (39%)	NA	NA
Factor V Leiden and prothrombin mutation analysis to assess the likelihood of thrombophilia as a possible cause of recurrent pregnancy loss.	22/36 (61%)	9/36 (25%)	4/36 (11%)	1/36 (3%)
Use of the CVD Multi-gene test to identify cardiovascular disease subtypes caused by a combination of genetic and environmental risk factors for targeted treatment.	20/36 (55%)	11/36 (31%)	3/36 (8%)	2/36 (6%)
HFE gene mutation testing to identify a genetic predisposition to iron overload causing hereditary haemochromatosis (HH) in patients with high serum iron stores.	20/36 (55%)	11/36 (31%)	2/36 (6%)	3/36 (8%)
**A *pathology supported genetic test* including components of the above assays as part of a wellness program that may be offered by medical schemes.**	**22/36 (61%)**	**6/36 (17%)**	**5/36 (14%)**	**3/36 (8%)**

Supplementary table 3: Section D results

**SECTION D:**			
**1=agree; 2=neutral; 3=disagree**	**1**	**2**	**3**
It is good practice to ask your patients about their **family history** and make a note (genogram) of the information provided.	33/36 (92%)	1/36 (3%)	2/36 (6%)
A secure patient database in an online computer system could be useful to document the family and medical history of my patients for future reference.	31/36 (86%)	3/36 (8%)	2/36 (6%)
A patient's family history is a strong predictor of disease development.	30/36 (84%)	3/36 (8%)	3/36 (8%)
Genetic information is of more value as a treatment guide than as a diagnostic test.	14/36 (39%)	13/36 (36%)	9/36 (25%)
Genetic information is of more value as a diagnostic test than as a treatment guide.	12/36 (33%)	11/36 (31%)	13/36 (36%)
**A genetic test is most meaningful when it can be used in a clinical context to simultaneously 1) diagnose treatable disease subtypes, 2) prevent cumulative risk, and 3) formulate an individualized treatment plan.**	**29/36 (80%)**	**5/36 (14%)**	**2/36 (6%)**
A regularly updated secure online computer program is useful to assist with selection of the appropriate genetic test(s) for your patient.	27/36 (75%)	7/36 (19%)	2/36 (6%)
Many medical aids pay for genetic testing.	5/36 (14%)	16/36 (44%)	15/36 (42%)
A secure online computer program to instantly generate a test quote with medical aid tariffs and a motivation based on the ICD-10 code provided would be very useful to clinicians and patients.	27/36 (75%)	6/36 (17%)	3/36 (8%)
Genetic testing is an unnecessary expense.	5/36 (14%)	9/36 (25%)	22/36 (61%)

Supplementary figure 1: The full genetic testing questionnaire

GENETIC TESTING QUESTIONNAIRE

Research Project Registration Number: HREC 25596, **N22/07/082**
Linked to previous study N09/08/224

**SCOPE OF SURVEY**: This survey aims to assess the attitudes and **needs of healthcare practitioners** towards the use of molecular genetic testing in clinical practice.

INITIATIVE: Stellenbosch University (SU) uses an integrated service-and research approach to develop **pathology supported genetic tests**. This means that a genetic profile is correlated with the patient's clinical history and pathology to assess gene expression and response to treatment. We would appreciate your help in conducting this survey to determine the attitudes and needs of healthcare practitioners for genetic testing in clinical practice.

CONFIDENTIALITY: The information provided will be used in congress presentations and for publication purposes without revealing your identity.

By completing this survey, you give consent for the use of the survey to determine physician knowledge and attitudes towards molecular genetic testing (e.g., relating to different test types, access to online counseling and test request service, new developments relating to a "lab-on-a-chip/point-of-care test kit" for DNA-based vs pathology/immunology/virology tests) and the application of such knowledge in public awareness campaigns, routine private practice or through Medical Schemes and Executive Health Screens. This information may be used towards research publications and development of further research strategies, should key knowledge gaps be identified in the readiness of healthcare practitioners to implement personalized genomic medicine in their daily clinical practice. The data captured in this survey may be shared with other researchers and be stored indefinitely.

I Agree to the above. YES ☐ NO ☐

Have you completed this questionnaire previously? YES ☐ NO ☐

**Thank you very much for your participation.**

YOUR DEMOGRAPHICS: Please tick appropriate boxes

Gender: ☐ Male ☐ Female

Age (years): ☐ 25 – 34 ☐ 35 – 44 ☐ 45 – 54 ☐ 55 – 64 ☐ 65 and older

Field of profession:

☐ Cardiologist ☐ Dietician ☐ GP ☐ Gynaecologist ☐ Oncologist ☐ Surgeon

☐ Other - please specify: . . . .

Number of years in clinical practice: ☐ Less than 5 ☐ 5 – 10 ☐ 11 – 20 ☐ more than 20

Please choose ONE of the following five options to rate your opinion of the statements below:					
**1=strongly agree; 2=agree; 3=neutral; 4=disagree; 5=strongly disagree**	**1**	**2**	**3**	**4**	**5**
There is a shortage of genetic counsellors and clinicians trained in genetics.					
The increase in number and complexity of genetic tests necessitates regular educational updates for clinicians.					
Personalised medicine refers to the ability to identify subgroups of patients that differ in their genetic susceptibility to a particular disease and/or their response to a specific intervention.					
Genetic alterations are permanent, but their expression can sometimes be modified by environmental factors (e.g. diet, medication) to increase or decrease risk.					
The current model of molecular genetics as a stand-alone laboratory service can be improved by integration with other health disciplines.					
The offer of genetic tests providing health-related information in the absence of clinical indications and individualised medical supervision may compromise patient health.					

Please choose ONE of the following five options to indicate whether you believe your patients could benefit from genetic testing performed in a clinical context:					
**1=strongly agree; 2=agree; 3=neutral; 4=disagree; 5=strongly disagree**	**1**	**2**	**3**	**4**	**5**
To diagnose a genetic disorder.					
To assess disease prognosis and recurrence risk.					
To identify genetic risk factors contributing to disease development.					
To prevent disease development in healthy individuals based on the family history.					
To identify subtypes of complex diseases to select the best treatment option.					
To identify high risk individuals who require aggressive treatment.					
To identify individuals with comorbidities at higher risk for infectious diseases					
To identify individuals most likely to benefit from diet and lifestyle changes only.					
To obtain information relating to more than one of the above in a single test.					

	**Please choose ONE of the following six options to indicate the clinical usefulness of the selected examples of genetic tests:**					
**1=strongly agree; 2=agree; 3=neutral; 4=disagree; 5=strongly disagree; 6=do not know this test**	**1**	**2**	**3**	**4**	**5**	**6**
BRCA1/2 gene screening to identify a high risk for familial breast cancer.						
Identification of a *BRCA1/2* mutation to motivate the need for a (prophylactic) bilateral mastectomy in women who developed breast cancer at a young age.						
The MammaPrint test to reduce the use of chemotherapy in patients with early-stage, oestrogen-positive breast cancer.						
CYP2D6 genotyping to identify Tamoxifen resistance in breast cancer patients.						
CYP2D6 genotyping to prevent side effects in 10-20% of individuals using anti-depressants and other commonly prescribed medications.						
Mutation analysis of the *LDL* receptor and/or *ApoE* genes in patients with high cholesterol to choose the best treatment strategy.						
Factor V Leiden and prothrombin mutation analysis to assess the likelihood of thrombophilia as a possible cause of recurrent pregnancy loss.						
Use of the CVD Multi-gene test to identify cardiovascular disease subtypes caused by a combination of genetic and environmental risk factors for targeted treatment.						
HFE gene mutation testing to identify a genetic predisposition to iron overload causing hereditary haemochromatosis (HH) in patients with high serum iron stores.						
A *pathology supported genetic test* including components of the above assays as part of a wellness program that may be offered by medical schemes.						

**Please choose ONE of the following five options to rate your opinion of the statements below:**					
**1=strongly agree; 2=agree; 3=neutral; 4=disagree; 5=strongly disagree**	**1**	**2**	**3**	**4**	**5**
It is good practice to ask your patients about their **family history** and make a note (genogram) of the information provided.					
A secure patient database in an online computer system could be useful to document the family and medical history of my patients for future reference.					
A patient's family history is a strong predictor of disease development.					
Genetic information is of more value as a treatment guide than as a diagnostic test.					
Genetic information is of more value as a diagnostic test than as a treatment guide.					
A genetic test is most meaningful when it can be used in a clinical context to simultaneously 1) diagnose treatable disease subtypes, 2) prevent cumulative risk, and 3) formulate an individualised treatment plan.					
A regularly updated secure online computer program is useful to assist with selection of the appropriate genetic test(s) for your patient.					

Many medical aids pay for genetic testing.					
A secure online computer program to instantly generate a test quote with medical aid tariffs and a motivation based on the ICD-10 code provided would be very useful to clinicians and patients.					
Genetic testing is an unnecessary expense.					

**The following questions will assess the use of genetic testing and counselling:**	**YES**	**NO**
Have you requested genetic testing for your patients in the past?		
Have your patients benefited (e.g., early diagnosis) from genetic testing in the past?		
Will you request genetic testing performed at an accredited laboratory for your patients in future?		
Have you referred patients for genetic counselling in the past to enable them to make their own free decisions about genetic testing?		
Will you refer patients for counselling by a registered genetic counsellor in future?		
Will you prefer to use a secure online computer program that provides all the relevant information to request genetic testing and/or counselling for your patients?		
Do you feel like you have sufficient genetic knowledge to request genetic tests for your patients?		
Would you be interested in workshops aimed at improving your genetic knowledge?		
Would you be interested in following social media platforms with regards to the latest genetic developments?		
Please indicate which platforms you are more likely to use:<br>Facebook<br>Instagram<br>Twitter<br>Other<br>If other, please specify:		
If more information is required, please specify:		
